# Supplementary material for: Stability of iron-bearing carbonates in the deep Earth’s interior
Source: Nat Commun. 2017 Jul 19;8:15960. doi: 10.1038/ncomms15960 (PMC5524932; doi:10.1038/ncomms15960)
Supplement: Supplementary Information [file ncomms15960-s1.pdf]

File Name: Supplementary Information
Description: Supplementary Figures, Supplementary Tables, Supplementary Notes and Supplementary References

File name: Peer Review File
Description:

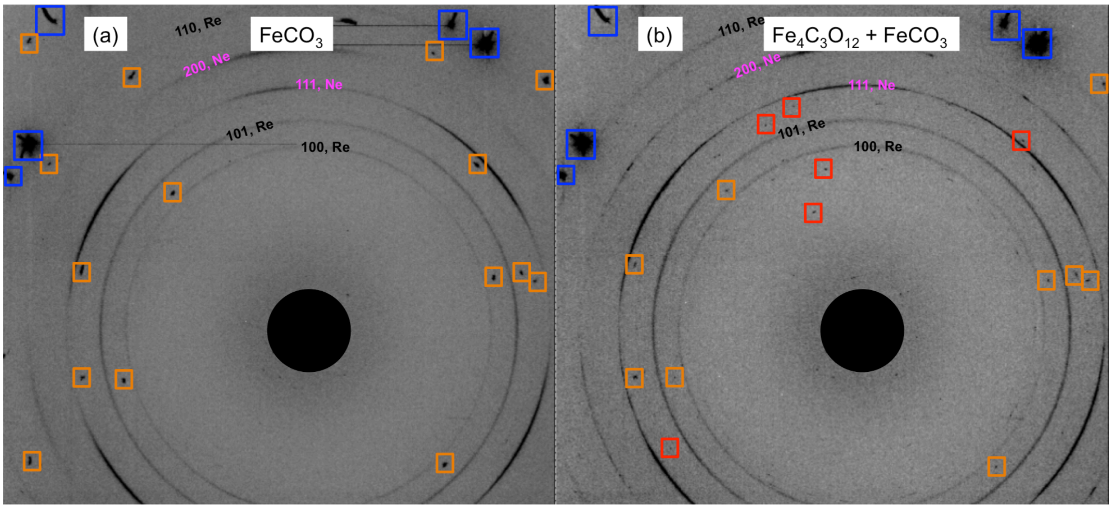

Supplementary Figure 1. Selected 2-D diffraction images of $FeCO_3$ before- and $FeCO_3$ + $Fe_4C_3O_{12}$ after- heating. Single crystal diffraction images were collected at 109(2) GPa at (a) ambient temperature before heating and (b) after heating at 1650(100) K. (b) Intensity loss of $FeCO_3$ single crystal spots (orange squares) and the appearance of new peaks indicate the transformation of $FeCO_3$ to $Fe_4C_3O_{12}$ (red squares). Blue squares indicate diamond diffraction spots from the diamond anvils. Numbers designate diffraction lines of neon pressure transmitting medium (magenta) and rhenium (black).

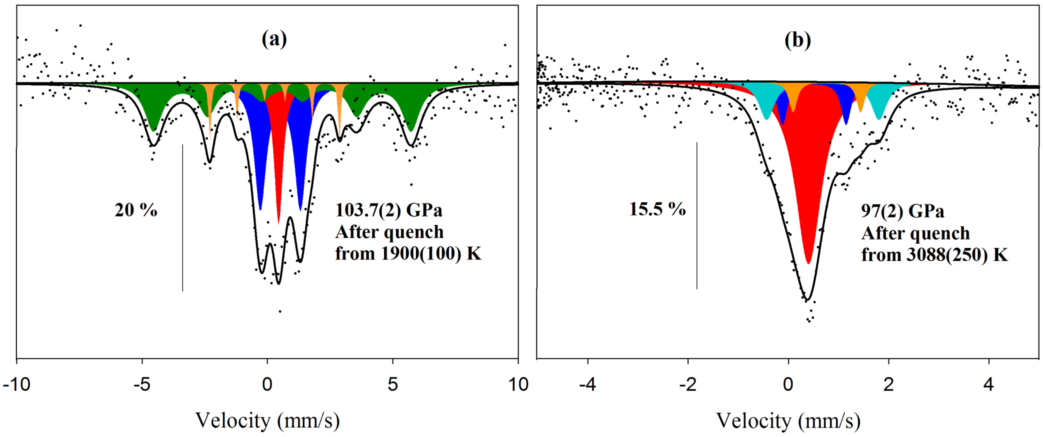

Supplementary Figure 2 | Mössbauer spectra of the run products after heating of $FeCO_3$ at high *P-T*. The spectra were collected at (*a*) 103.7(2) GPa after heating at 1900(100) K, and (*b*) at 97(2) GPa after heating at 3088(250) K. The hyperfine parameters for each component are reported in Table S4. The spectra are fitted with full transmission integral and are shown by continues lines. In percentage on the left of each spectrum the relative absorption.

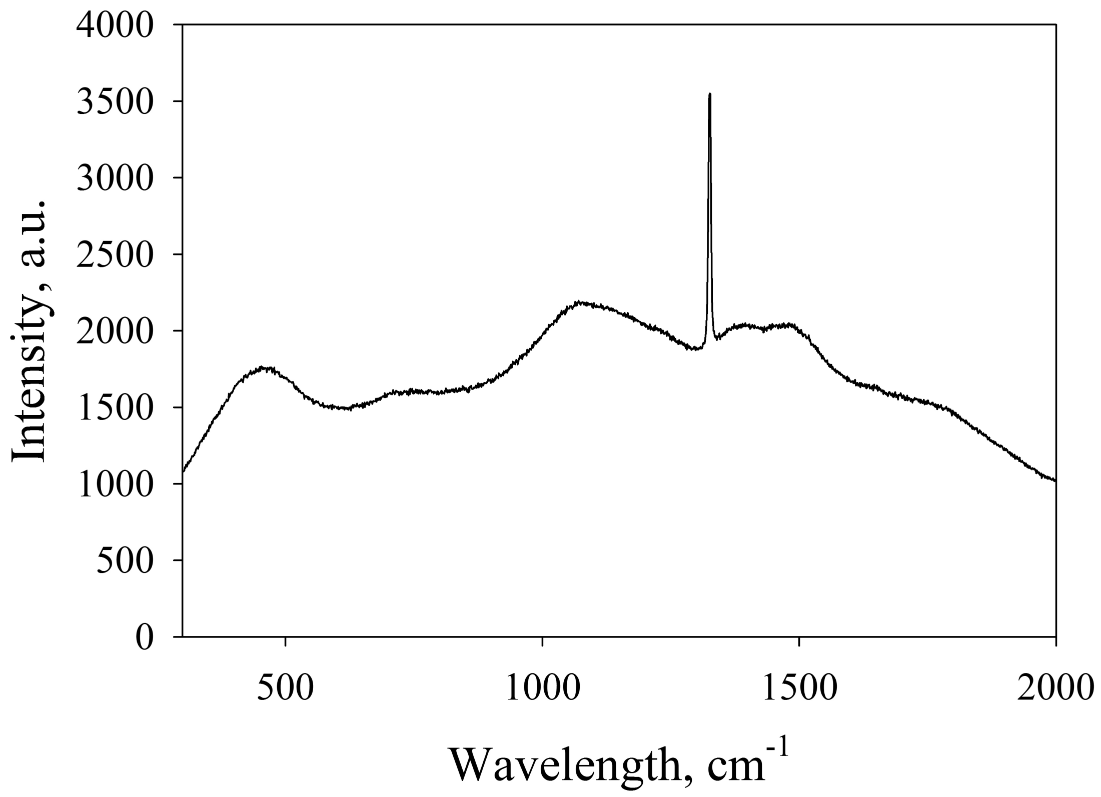

Supplementary Figure 3 | Nano-diamond Raman spectrum collected after heating $FeCO_3$ at 78(1) GPa and 1950(100) K. Raman spectrum collected after decompression to ambient conditions of the sample laser heated at 78(1) GPa and 1950(100) K. Starting material was pure $FeCO_3$ loaded in Ne pressure transmitting medium. After decompression sample was removed from pressure chamber and placed on glass plate. Strong broad features are typical for nano-diamond and band at ~1330 cm^{-1} characteristic for diamond.

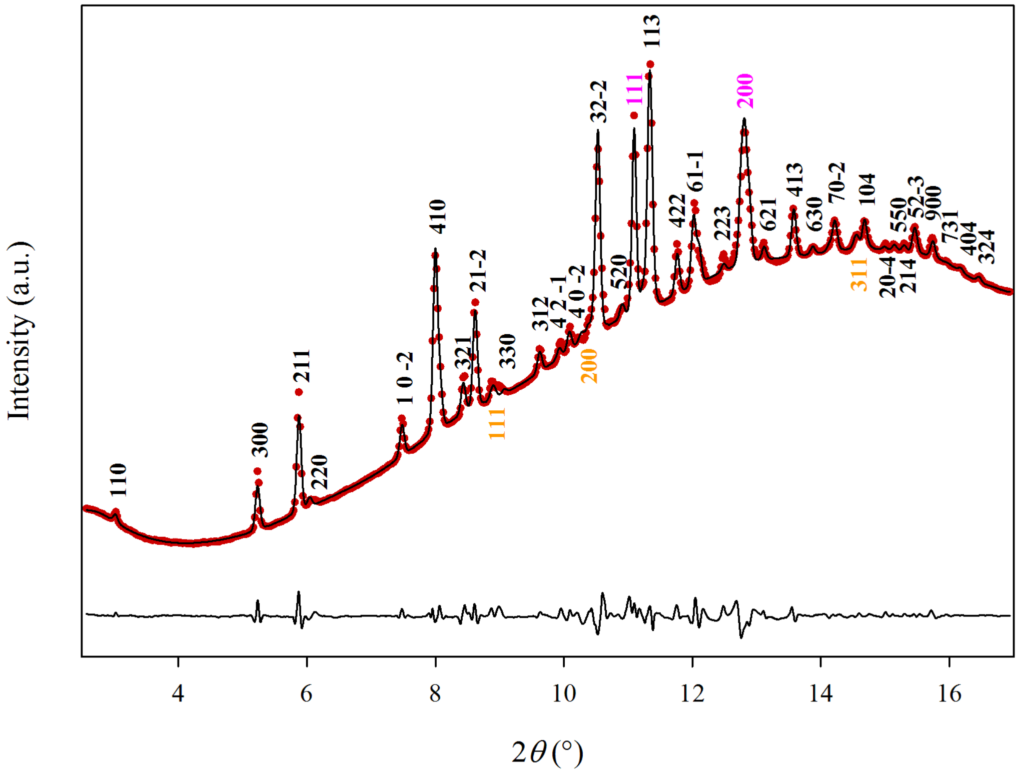

Supplementary Figure 4 | Rietveld refinement of an X-Ray diffraction pattern provided by Liu *et al.*[13] of tetrairon (III) orthocarbonate at 90 GPa and room temperature. The pattern was collected after heating magnesio-siderite $(Mg_{0.35}Fe_{0.65})CO_3$ at 90 GPa and 2200 K. The high-pressure carbonate was fitted using our model of tetrairon (III) orthocarbonate (Table S1). Red dots: measured powder diffraction pattern; black solid line: refined profile; black solid line (bottom): residual between the observation and the refinement. Indices of $(Mg,Fe)_4C_3O_{12}$ in black, Ne in purple, and Au in orange. The refinement was performed by using GSAS package.

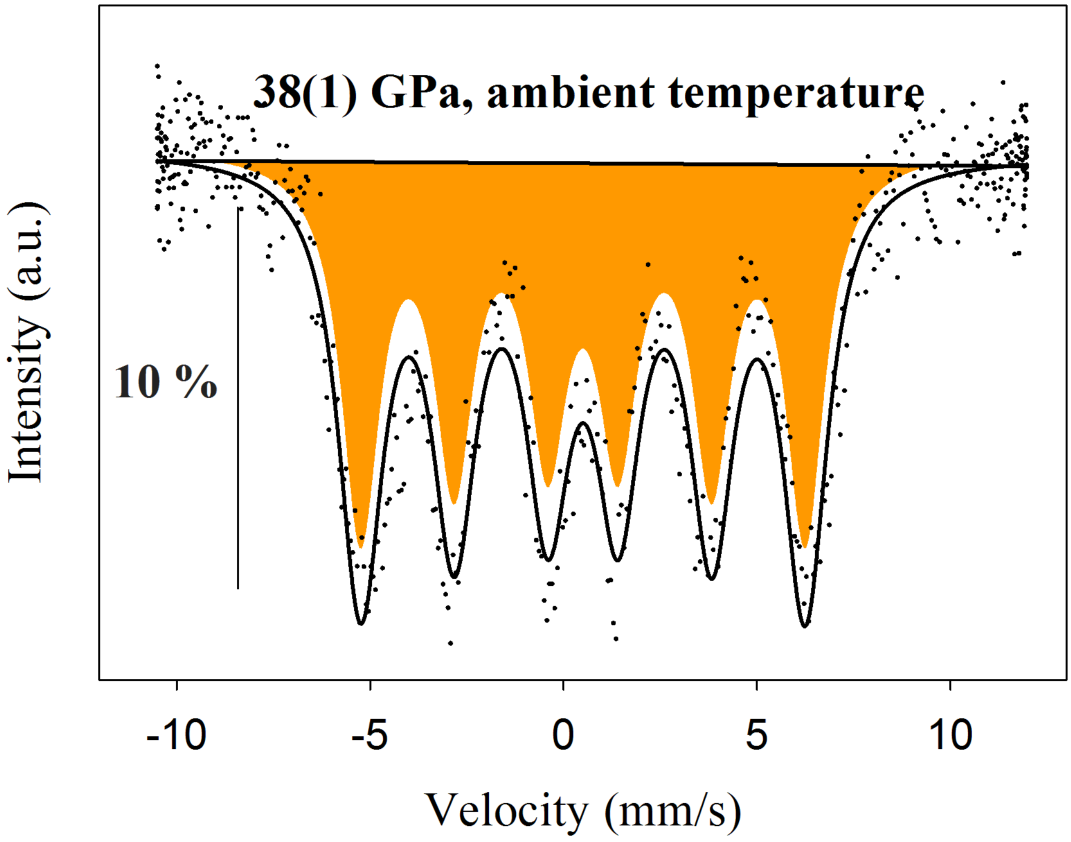

Supplementary Figure 5 | Mössbauer spectra of pure HP-Fe_3O_4 at 38(1) GPa and ambient temperature. The hyperfine parameters are reported in Table S4. The data are fitted using a subspectrum (orange) that does not add up to the total spectrum due to the properties of the full transmission integral fit. In percentage on the left of the spectrum the relative absorption.

Supplementary Table 1. Experimental runs *PT* path and products obtained at the specific conditions

Experiment	P (GPa)*	T (K)	Observed phases	Comments	Analytical Technique
1. ID09a (ESRF)	5(1)	293	$FeCO_3$		SXRD
	19.5(1)	293	$FeCO_3$		SXRD
	29.5(1)	293	$FeCO_3$		SXRD
	39.6(1)	293	$FeCO_3$		SXRD
	49.4(1)	293	$FeCO_3$		SXRD
2. ID18 (ESRF)	25.2(1)	2050(150)	$FeCO_3$, α-Fe_2O_3		MS
3. ID27 (ESRF)	28.7(1)	293	$FeCO_3$		SXRD
		1550(100)	$FeCO_3$		SXRD
		2250(150)	$FeCO_3$, HP-Fe_3O_4		SXRD
4. ID18 (ESRF)	30.8(1)	2150(150)	$FeCO_3$, HP-Fe_3O_4		MS
5. ID09a (ESRF)	37(1)	2500(150)	$FeCO_3$, HP-Fe_3O_4		SXRD
6. ID18 (ESRF)	39(1)	2000(150)	$FeCO_3$, HP-Fe_3O_4		MS
7. ID18 (ESRF)	40(1)	393(40)	$FeCO_3$		MS
		464(45)	$FeCO_3$		MS
8. ID18 (ESRF)	45(1)	293	$FeCO_3$		MS
		570(50)	$FeCO_3$		MS
9. ID09a (ESRF)	50(1)	1750(100)	$FeCO_3$		SXRD
		2350(150)	$FeCO_3$, HP-Fe_3O_4		SXRD
		2450(150)	$FeCO_3$, HP-Fe_3O_4		SXRD
10. ID18 (ESRF)	52.5(1)	293	$FeCO_3$		MS
		430(40)	$FeCO_3$		MS
11. 13-IDD (APS)	69.3(1)	1187(100)	$FeCO_3$		SXRD
	70.7(1)	1257(100)	$FeCO_3$		SXRD
		1413(100)	$FeCO_3$		SXRD
12. ID18, ID27 (ESRF)	74(1)	1750(100)	$Fe_4C_3O_{12}$, $Fe_4C_4O_{13}$		SXRD
13. ID18, ID27 (ESRF)	75(1)	2200(150)	$Fe_4C_3O_{12}$, $Fe_4C_4O_{13}$, HP-Fe_3O_4, ppv-Fe_2O_3, Fe_5O_7		SXRD
14. ID27 (ESRF)	91(2)	2500(150)	$Fe_4C_4O_{13}$, HP-Fe_3O_4		SXRD
15. 13-IDD (APS)	97(2)	3088(250)	$Fe_4C_4O_{13}$		SXRD
16. ID18 (ESRF)	97(2)	293	$Fe_4C_4O_{13}$, HP-Fe_3O_4	Temperature quenched after heating at 97(2) GPa and 3090	MS
17. ID09a	110(2)	1150(100)	$FeCO_3$	Experiment 17 - XRSD was collected in situ while heating	SXRD
		1350(100)	$FeCO_3$	corresponding temperatures	SXRD
		1400(100)	$Fe_4C_3O_{12}$		SXRD
		1500(100)	$Fe_4C_3O_{12}$		SXRD
		1550(100)	$Fe_4C_3O_{12}$, $Fe_4C_4O_{13}$		SXRD
		1650(100)	$Fe_4C_3O_{12}$, $Fe_4C_4O_{13}$		SXRD
		1700(100)	$Fe_4C_3O_{12}$, $Fe_4C_4O_{13}$		SXRD
		1750(100)	$Fe_4C_3O_{12}$, $Fe_4C_4O_{13}$		SXRD
		1900(100)	$Fe_4C_3O_{12}$, $Fe_{13}O_{19}$		SXRD
18. ID18 (ESRF)	103.7(2)	293	$Fe_4C_3O_{12}$, $Fe_{13}O_{19}$	Temperature quenched after heating at 110(2) GPa and 190	MS

SXRD, Single crystal X-ray Diffraction; MS, Mössbauer Spectroscopy

*Pressures are indicative for ambient temperature or temperature-quenched samples

Supplementary Table 2. Details of crystal structure refinements of *HP*-carbonates and atomic coordinates

**Crystallographic data**	$Fe_4C_3O_{12}$	$Fe_4C_4O_{13}$
***P, T* conditions of XRD experiment**	74(1) GPa<br>after heating at 1750(100) K	97(2) GPa<br>after heating at 3088(250) K
**Crystal system**	Trigonal	Monoclinic
**Space group**	R 3 c	C 1 2/c 1
***a* (Å)**	12.762(2)	10.261(3)
**b (Å)**	12.762(2)	3.985(3)
***c* (Å)**	5.332(1)	13.455(5)
**α (°)**	90	90
**β (°)**	90	107.85(4)
**γ (°)**	120	90
***V* ($Å^3$)**	752.0(3)	523.76(28)
Z	6	4
**Independent reflections / R_{int}**	386/0.0427	1503/0.0705
**Refinement method**	Full matrix least squares on F^2	Full matrix least squares on F^2
**Data / restraints / parameters**	386 / 1 / 33	346 / 0 / 53
**Goodness of fit on F^2**	1.125	1.214
**Final *R* indices [$I > 2\sigma(I)$] R_1 / wR_2**	0.0468/0.1133	0.03830.0964
**ICSD reference N**	432930	432931

$Fe_4C_3O_{12}$ - 74(1) GPa after heating at 1750(100) K

**Site**	**x**	**y**	**z**	**Uiso**
Fe(1)	1/3	2/3	0.0121(7)	0.0111(8)
Fe(2)	0.2274(2)	0.2282(2)	0.0468(5)	0.0468(5)
O(1)	0.2034(11)	0.0140(12)	0.017(3)	0.010(2)
O(2)	0.4119(9)	0.5391(9)	0.0342(19)	0.0094(18)
O(3)	0.3995(8)	0.3275(9)	0.029(2)	0.0084(18)
O(4)	0.2296(9)	0.3819(8)	0.036(2)	0.0087(17)
C(1)	0.1287(8)	0.0584(8)	0.100(2)	0.0095(17)

$Fe_4C_4O_{13}$ - 97(2) GPa after heating at 3088(250) K

**Site**	**x**	**y**	**z**	**Uiso**
Fe(1)	0.1242(1)	0.4730(2)	0.0936(1)	0.0119(5)
Fe(2)	0.1693(1)	0.4698(2)	0.8360(1)	0.0116(5)
O(1)	0.1855(5)	0.0342(8)	0.3799(4)	0.0107(8)
O(2)	0.0873(5)	0.3820(8)	0.4552(4)	0.0111(8)
O(3)	0.0742(4)	0.2853(8)	0.2048(4)	0.0095(8)
O(4)	0	0.2020(11)	0.7500(1)	0.0096(11)
O(5)	0.0007(5)	0.1162(9)	0.5893(4)	0.0112(8)
O(6)	0.1811(5)	0.0764(9)	0.0397(4)	0.0117(8)
O(7)	0.1876(4)	0.1948(8)	0.7120(3)	0.0094(8)
C(1)	0.1183(8)	0.0558(14)	0.4494(6)	0.0105(11)
C(2)	0.0695(8)	0.0315(12)	0.6912(6)	0.0123(12)

Supplementary Table 3. High-pressure carbonates selected geometric parameters (Å, °).

**$Fe_4C_3O_{12}$**				**$Fe_4C_4O_{13}$**			
74(1) GPa after heating at 1750(100) K				97(2) GPa after heating at 3088(250) K			
Fe(1) − O(4)	1.935(11)	O3 − C1 − O4	108.7(11)	Fe1 − O2	1.874(5)	O2 − C1 − O1	106.7(5)
Fe(1) − O(4)	1.935(11)	O2 − C1 − O3	119.2(12)	Fe1 − O3	1.879(5)	O6 − C1 − O1	114.7(6)
Fe(1) − O(4)	1.935(11)	O1 − C1 − O3	108.0(14)	Fe1 − O6	1.890(5)	O6 − C1 − O2	113.3(5)
Fe(1) − O(4)	1.966(10)	O4 − C1 − O1	114.4(14)	Fe1 − O1	1.904(5)	O6 − C1 − O5	106.5(6)
Fe(1) − O(4)	1.966(10)	O4 − C1 − O2	103.4(11)	Fe1 − O7	2.020(4)	O2 − C1 − O5	108.0(5)
Fe(1) − O(4)	1.966(10)	O1 − C1 − O2	101.8(10)	Fe1 − O5	2.060(4)	O1 − C1 − O5	107.3(6)
Fe(1) − O(1)	2.303(10)			Fe1 − O2	2.097(5)		
Fe(1) − O(1)	2.303(10)			Fe2 − O6	1.901(5)	O4 − C2 − O3	113.9(4)
Fe(1) − O(1)	2.303(10)			Fe2 − O7	1.989(4)	O4 − C2 − O7	103.5(6)
Fe(2) − O(4)	1.906(9)			Fe2 − O3	1.991(4)	O4 − C2 − O5	104.5(5)
Fe(2) − O(4)	1.912(9)			Fe2 − O7	2.052(4)	O3 − C2 − O5	111.8(5)
Fe(2) − O(2)	1.930(11)			Fe2 − O1	2.055(3)	O7 − C2 − O5	104.2(6)
Fe(2) − O(3)	1.948(9)			Fe2 − O1	2.065(3)	O3 − C2 − O7	117.6(4)
Fe(2) − O(1)	2.016(10)			Fe2 − O4	2.086(3)		
Fe(2) − O(1)	2.066(11)			Fe2 − O2	2.111(6)	C1 − O5 − C2	112.6(6)
Fe(2) − O(3)	2.073(11)			C1 − O6	1.299(8)	C2 − O4 − C2	121.6(5)
Fe(2) − O(3)	2.263(10)			C1 − O2	1.324(11)	C1 − C2 − C2	91.4(4)
				C1 − O1	1.346(7)		
C1 − O4	1.306(17)			C1 − O5	1.357(8)		
C1 − O1	1.349(17)			C2 − O3	1.275(6)		
C1 − O2	1.350(19)			C2 − O5	1.328(8)		
C1 − O3	1.402(17)			C2 − O4	1.378(8)		
				C2 − O7	1.394(9)		

Supplementary Table 4. Hyperfine parameters derived from room temperature SMS spectra of $FeCO_3$ and run products

Pressure (GPa)	Temperature (K)	Phase	Component/ Color in Figs. 3, S2 and S3	CS[a] mm/s	QS[b] mm/s	Area %	FWHM[c] mm/s	BHF[d] T
25.2(1)	before heating	$FeCO_3$	Fe^{2+} (HS) (blue)	1.09(2)	1.72(4)	100	0.55(9)	-
25.2(1)	quenched from	$FeCO_3$	Fe^{2+} (HS) (blue)	1.09(2)	1.77(4)	80(4)	0.53(5)	-
	2050(150)	α-Fe_2O_3	sextet (green)	0.34(5)	-	20(4)	0.24(17)	49.2(4)
30.8(1)	before heating	$FeCO_3$	Fe^{2+} (HS) (blue)	1.02(2)	1.77(4)	100	0.42(5)	-
30.8(1)	quenched from	$FeCO_3$	Fe^{2+} (HS) (blue)	1.01(1)	1.78(2)	68(4)	0.50(8)	-
	2100(150)	HP-Fe_3O_4	sextet (orange)	0.61(9(	-	32(4)	0.97(40)	33.8(7)
39(1)	before heating	$FeCO_3$	Fe^{2+} (HS) (blue)	0.99(2)	1.70(4)	100	0.51(11)	-
39(1)	quenched from	$FeCO_3$	Fe^{2+} (HS) (blue)	0.94(4)	1.70(7)	11(4)	0.21(17)	-
	2000(150)	HP-Fe_3O_4	sextet orange)	0.56(7)	-	89(4)	1.57(17)	37.5(5)
45(1)	before heating	$FeCO_3$	Fe^{2+} (HS) (blue)	0.94(4)	1.10(8)	43(16)	0.25(16)	-
			Fe^{2+} (LS) (red)	0.69(5)	-	57(16)	0.57(23)	-
45(1)	570(50), *in situ*	$FeCO_3$	Fe^{2+} (HS) (blue)	0.74(4)	1.25(7)	83(6)	0.40(9)	-
	heating		Fe^{2+} (LS) (red)	0.57(8)	-	17(6)	*0.30*	-
38(1)	before heating	HP-Fe_3O_4	sextet (orange)	0.50(3)	-	100.00	1.22(7)	35.6(2)
103.7(2)	quenched from		singlet (red)	0.41(1)	-	16(1)	0.63(6)	-
	1900(100)		doublet (blue)	0.48(1)	1.60(2)	36(2)	0.76(4)	-
			sextet (green)	0.53(1)	0	41(1)	0.93(4)	31.8(1)
			sextet (orange)	0.26(1)	0	6(1)	0.25(4)	15.8(1)
97(2)	3088(250)		singlet	0.40(1)	-	65(2)	0.64(2)	-
			doublet	0.52(1)	1.27(1)	12(1)	0.24(3)	-
			doublet	0.69(1)	2.24(2)	16(1)	0.37(3)	-
			doublet	0.77(1)	1.35(2)	7(1)	0.19(4)	-

[a] CS: center shift relative to α-Fe
[b] QS: quadrupole splitting
[c] FWHM: full width at half maximum including the source linewidth
[d]: BHF: magnetic hyperfine field

Supplementary Table 5. Examples of iron oxides phases found in products of decomposition of $FeCO_3$ treated at different P,T conditions. Phase identifications was based on the results of single crystal diffraction data.

Crystallographic data	$Fe_{13}O_{19}$	HP-Fe_3O_4	Fe_5O_7	ppv-Fe_2O_3
***P*, *T* conditions of XRD experiment**	103.7(2) GPa<br>after annealing at 1900(100) K	51(1) GPa<br>after annealing at 2350(150) K	74(1) GPa<br>after annealing at 2200(150) K	74(1) GPa<br>after annealing at 2200(150) K
**Crystal system**	Monoclinic	Orthorhombic	monoclinic	orthorhombic
**Space group**	C 1 2/m 1	C m c m	C 2/m	C m c m
***a* (Å)**	19.233(2)	2.673(1)	8.638(4)	2.631(1)
**b (Å)**	2.582(1)	9.150(9)	2.635(1)	8.585(2)
***c* (Å)**	9.550(11)	9.202(4)	7.977(3)	6.326(9)
**α (°)**	90	90	90	90
**β (°)**	118.39(3)	90	106.02(4)	90
**γ (°)**	90	90	90	90
**V (Å^3)**	417.2(5)	225.0(2)	174.56(15)	142.9(2)
**Z**	8	4	2	4
**Independent reflections / R_{int}**	415/0.0588	169/0.0474	231/0.0203	157/0.0096
**Refinement method**		Full matrix least squares on F^2		
**Data / restraints / parameters**	246 / 0 / 48	86 / 0 / 17	158 / 0 / 18	86 / 0 / 14
**Final *R* indiced [$I > 2\sigma(I)$ R_1 / wR_2**	0.1273/0.0780	0.0796/0.1914	0.0672/0.1635	0.0938/0.2189
**ICSD reference N**	238769	430561	430563	430559

Supplementary Note 1. Mössbauer spectra of high pressure carbonates

We collected SMS spectra for the run products after heating of $FeCO_3$ at 103.7(2) GPa and 1900(100) K and at 97(2) GPa and 3088(250) K (Supplementary Figure. 2). From XRSD analyses performed on the same samples, we identified the products to be mixtures of $Fe_4C_3O_{12}$ + $Fe_{13}O_{19}$ + unreacted $FeCO_3$ at 103.7(2) (Supplementary Figure 2a, Supplementary Table 5) GPa and $Fe_4C_4O_{13}$ + unreacted $FeCO_3$ at 97(2) GPa (Supplementary Figure 2b, Supplementary Table 5). Unfortunately, for such multicomponent mixtures it is impossible to propose unique Mössbauer fitting models. However, we can state that all components except one have hyperfine parameters (particularly CS) compatible with iron's in HS rather than LS states (Supplementary Table 4), which is in perfect agreement wit the structural data (Fe-O bonding lengths in both *HP*-carbonates structures, Supplementary Table 2). Only the red singlets (Supplementary Figure 2a and 2b) can be almost surely addressed to LS-Fe^{2+} in $FeCO_3$ due to their CS values 0.41(1) (Supplementary Figure 2a) and 0.40(1) (Supplementary Figure 2b), which are in perfect agreement to those expected for LS-Fe^{2+} in siderite at those conditions[17].

Supplementary Note 2. Bond Angle Variance

Cation coordination polyhedral in most ionic structures only approximate to regular geometrical forms. Deviation from regularity can be partly characterized by using distortion parameters. In order to better describe the geometry of the tetrahedral coordination complex, we measured the bond-angle distortion (or angle variance) for the CO_4 groups in the orthocarbonate. The bond angle distortion $\sigma(\text{tet})^2$ is dimensionless and it is a convenient and realistic measure of distortion for those polyhedral that show variations in both bond angle and bond length. It gives a quantitative measure of the polyhedral distortion, which is independent of the effective size of the polyhedron[1]. When the angle variance is equal to 0 the described polyhedron is undistorted

For tetrahedral complexes and coordination polyhedra one can measure the variance parameter (σ^2) using the following equation[1]:

$$\sigma^2_{(tet)} = \sum_{i=1}^{6} (\theta_i - \theta_0)^2 / 5 \qquad (1)$$

where, θ_0 is the ideal bond angle for a regular polyhedron (e.g. 109.47° for a tetrahedron) and θ_i is the *i*th bond angle.

Supplementary References

[1] Robinson, K. Gibbs, G.V. & Ribbe, P.H. Quadratic elongation: A quantitative measure of distortion in coordination polyhedral. *Science* **172**, 567-570 (1971).
